# Supplementary material for: Associations of Allopregnanolone and Related Steroid Hormones with Prenatal Psychosocial Distress in the Healthy Start Cohort
Source: medRxiv. 2026 Jul 24:2026.07.22.26358684. Preprint. [Version 1] doi: 10.64898/2026.07.22.26358684 (PMC13419650; doi:10.64898/2026.07.22.26358684)
Supplement: Supplementary file 1 [file media-1.pdf]

Supplemental Material

Title: Associations of Allopregnanolone and Related Steroid Hormones with Prenatal Psychosocial Distress in the Healthy Start Cohort

Authors: Gabriella Mayne[1], MA, K. Joseph Hurt[2] MD PhD, Sara Yeatman, PhD[1], Jost Klawitter, PhD[3], David Tracer, PhD[1], Uwe Christians, MD, PhD[3], Dana Dabelea, MD, PhD[4,5], and Wei Perng, PhD[4]

Affiliations:

1. Department of Health & Behavioral Sciences, University of Colorado, Denver, CO, 80204 USA
2. Divisions of Maternal Fetal Medicine and Reproductive Sciences, Department of Obstetrics and Gynecology, University of Colorado Anschutz Medical Campus, Aurora, Colorado 80045, USA
3. iC42 Clinical Research & Development, Department of Anesthesiology, University of Colorado Anschutz Medical Campus, Aurora, CO 80045, USA
4. Lifecourse Epidemiology of Adiposity and Diabetes (LEAD) Center and the Department of Epidemiology, Colorado School of Public Health, University of Colorado Anschutz Medical Campus, Aurora, CO 80045, USA
5. Department of Pediatrics, School of Medicine, University of Colorado Anschutz Medical Campus, Aurora, Colorado 80045, USA.

***Corresponding author:**

Gabriella Mayne, MA
University of Colorado, Denver
Department of Health and Behavioral Sciences
1201 Larimer Street
Denver, CO 80204, USA
gabriella.2.mayne@ucdenver.edu
ORCID ID: 0000-0001-9860-2939

SUPPLEMENTAL MATERIAL:

Supplemental Table S1. Bivariate associations of maternal sociodemographic and prenatal characteristics comparing the participants included in this study (n=237) to those Healthy Start participants not included (n=1,173).

	**Included**	**Not Included**	***P*-value[a]**
	**n = 237<br>mean ± SD or N (%)**	**n = 1,173<br>mean ± SD or N (%)**	
**Sociodemographic characteristics**			
Age (years)	29 ± 6	28 ± 6	**<0.001**
Nulliparous	91 (38)	744 (67)	**<0.001**
Married/cohabiting	199 (84)	931 (80)	0.20
Use of public assistance program	72 (31)	348 (39)	**0.02**
Maternal education level			**0.01**
<High school	32 (14)	172 (15)	
High school degree or GED	32 (14)	227 (19)	
Some college or associate's degree	45 (19)	289 (25)	
College graduate	64 (27)	245 (21)	
Graduate degree	64 (27)	240 (20)	
Annual household income ≥$70,000	107 (54)	353 (38)	**<0.001**
Race/ethnicity			**<0.001**
Non-Hispanic White	143 (60)	281 (43)	
Hispanic	54 (23)	270 (42)	
Non-Hispanic Black	26 (11)	60 (9.2)	
Non-Hispanic Other[b]	14 (5.9)	39 (6.0)	
Geographic region			**0.02**
Urban	219 (96)	955 (99)	
Rural	8 (3.5)	11 (1.1)	
Born in US	200 (84)	1,008 (86)	0.50
**Prenatal characteristics**			
Pre-pregnancy BMI, kg/m^2	25.4 ± 6	25.6 ± 6	0.30
Fetus is female	115 (49)	535 (48)	0.90
Gestational diabetes mellitus	11 (5.0)	44 (4.2)	0.90
Gestational hypertension	16 (7.0)	73 (6.6)	0.90
Anemia	44 (19)	226 (20)	0.60

Preeclampsia	11 (4.8)	40 (3.6)	0.40
Smoked during pregnancy	12 (5.1)	90 (7.7)	0.20
Psychiatric disorder	38 (16)	204 (18)	0.50
Use of anti-depressant/anxiety medication during pregnancy	13 (5.5)	57 (4.9)	0.50

Data presented as means ± standard deviations or N (%). Bolded values indicate statistical significance at alpha = 0.05.
[a]*P*-values computed by Chi-square or Student T-tests.
[b]Other includes individuals identifying as American Indian or Alaska Native, Hawaiian or Pacific Islander, Asian, or mixed race.

Supplemental Table S2. Effect estimates for high-distress status (β [95% CI] and % Difference [95% CI]) across various sensitivity analyses for allopregnanolone (ALLO)

**Sensitivity Analysis**	**β (95% CI)**	**% Difference (95% CI)**	***P*-value**
ALLO Model 1 (gestational age at blood draw, maternal age, fetal sex, prenatal smoking)	-0.23 (-0.37, -0.09)	-20.57 (-30.87, -8.74)	**<0.001**
ALLO Model 1 + Pre-pregnancy BMI (kg/m^2)	-0.22 (-0.34, -0.10)	-19.71 (-28.84, -9.41)	**<0.001**
ALLO Model 1 + Preeclampsia	-0.24 (-0.39, -0.10)	-21.66 (-31.96, -9.81)	**<0.001**
ALLO Model 1 + Anti-depressant/anxiety medication	-0.21 (-0.35, -0.07)	-18.83 (-29.57, -6.44)	**0.004**

Supplemental Table S3. Comparison of high-distress effect estimates (β and %Difference) for all models between the full cohort vs exclusion of the outlier for ALLO, progesterone, and pregnanolone.

Biomarker or Ratio	Model	Cohort	β (log) [95% CI]	% Difference [95% CI]
Allopregnanolone (ALLO)	Unadjusted	Full cohort	-0.23 (-0.36, -0.10)	-20.3 (-29.9, -9.3)
		No-outlier	-0.22 (-0.35, -0.10)	-20 (-29.7, -9.1)
	Model 1	Full cohort	-0.23 (-0.37, -0.09)	-20.6 (-30.9, -8.7)
		No-outlier	-0.23 (-0.37, -0.09)	-20.3 (-30.6, -8.5)
	Model 2	Full cohort	-0.13 (-0.3, 0.01)	-12.4 (-24.4, 1.4)
		No-outlier	-0.13 (-0.28, 0.02)	-12.1 (-24, 1.7)
	Model 3	Full cohort	-0.12 (-0.26, 0.03)	-10.9 (-22.9, 3.1)
		No-outlier	-0.11 (-0.26, 0.03)	-10.5 (-22.5, 3.4)
Progesterone	Unadjusted	Full cohort	-0.02 (-0.09, 0.05)	-2 (-8.9, 5.4)
		No-outlier	-0.02 (-0.09, 0.05)	-1.8 (-8.7, 5.5)
	Model 1	Full cohort	0.01 (-0.07, 0.09)	1 (-6.6, 9.3)
		No-outlier	0.01 (-0.07, 0.09)	1.1 (-6.5, 9.3)
	Model 2	Full cohort	0.03 (-0.06, 0.11)	3 (-5.3, 12.1)
		No-outlier	0.03 (-0.05, 0.11)	3 (-5.2, 12)

	Model 3	Full cohort	0.03 (-0.06, 0.11)	2.7 (-5.6, 11.8)
		No-outlier	0.03 (-0.06, 0.11)	2.8 (-5.5, 11.8)
Pregnanolone	Unadjusted	Full cohort	-0.20 (-0.37, -0.03)	-17.9 (-30.6, -2.8)
		No-outlier	-0.19 (-0.36, -0.03)	-17.6 (-30.3, -2.7)
	Model 1	Full cohort	-0.12 (-0.30, 0.06)	-11.4 (-26.1, 6.3)
		No-outlier	-0.12 (-0.30, 0.06)	-11.2 (-25.9, 6.3)
	Model 2	Full cohort	-0.02 (-0.22, 0.17)	-2.2 (-19.4, 18.7)
		No-outlier	-0.02 (-0.21, 0.17)	-2.1 (-19.2, 18.6)
	Model 3	Full cohort	-0.01 (-0.20, 0.19)	-0.7 (-18.2, 20.6)
		No-outlier	-0.01 (-0.20, 0.19)	-0.7 (-18, 20.4)
Cortisol	Unadjusted	Full cohort	-0.05 (-0.12, 0.03)	-4.5 (-11.5, 3)
		No-outlier	-0.05 (-0.12, 0.03)	-4.6 (-11.6, 2.9)
	Model 1	Full cohort	-0.05 (-0.14, 0.03)	-5.1 (-12.6, 3)
		No-outlier	-0.05 (-0.14, 0.03)	-5.2 (-12.7, 3)
	Model 2	Full cohort	0.00 (-0.09, 0.09)	0.1 (-8.2, 9.1)
		No-outlier	0.00 (-0.09, 0.09)	0.1 (-8.2, 9.1)
	Model 3	Full cohort	0.02 (-0.07, 0.10)	1.7 (-6.7, 10.7)

		No-outlier	0.02 (-0.07, 0.10)	1.6 (-6.7, 10.7)
Cortisone	Unadjusted	Full cohort	0.04 (-0.02, 0.09)	3.8 (-1.7, 9.6)
		No-outlier	0.04 (-0.02, 0.09)	3.9 (-1.6, 9.6)
	Model 1	Full cohort	0.03 (-0.03, 0.09)	3.2 (-2.7, 9.4)
		No-outlier	0.03 (-0.03, 0.09)	3.2 (-2.7, 9.4)
	Model 2	Full cohort	0.04 (-0.03, 0.1)	3.7 (-2.7, 10.5)
		No-outlier	0.04 (-0.03, 0.1)	3.7 (-2.6, 10.5)
	Model 3	Full cohort	0.04 (-0.02, 0.10)	4.1 (-2.4, 10.9)
		No-outlier	0.04 (-0.02, 0.10)	4.1 (-2.4, 10.9)
ALLO-to-Progesterone	Unadjusted	Full cohort	-0.20 (-0.31, -0.09)	-18.1 (-26.4, -9)
		No-outlier	-0.20 (-0.31, -0.09)	-18 (-26.3, -8.8)
	Model 1	Full cohort	-0.23 (-0.35, -0.12)	-20.7 (-29.3, -11.1)
		No-outlier	-0.23 (-0.35, -0.12)	-20.6 (-29.2, -11)
	Model 2	Full cohort	-0.15 (-0.27, -0.03)	-14.1 (-23.9, -3)
		No-outlier	-0.15 (-0.27, -0.03)	-14 (-23.7, -2.9)
	Model 3	Full cohort	-0.13 (-0.25, -0.012)	-12.2 (-22, -1.2)
		No-outlier	-0.13 (-0.25, -0.011)	-12.1 (-21.8, -1.1)

ALLO-to-Pregnanolone	Unadjusted	Full cohort	-0.01 (-0.14, 0.12)	-1.4 (-12.8, 11.4)
		No-outlier	-0.02 (-0.14, 0.12)	-1.5 (-12.8, 11.4)
	Model 1	Full cohort	-0.09 (-0.22, 0.04)	-8.3 (-19.4, 4.3)
		No-outlier	-0.09 (-0.22, 0.04)	-8.3 (-19.4, 4.3)
	Model 2	Full cohort	-0.08 (-0.22, 0.06)	-8 (-19.9, 5.6)
		No-outlier	-0.08 (-0.22, 0.06)	-8 (-19.9, 5.6)
	Model 3	Full cohort	-0.08 (-0.22, 0.06)	-7.7 (-19.7, 6.2)
		No-outlier	-0.08 (-0.22, 0.06)	-7.7 (-19.7, 6.2)
ALLO-to-Cortisol	Unadjusted	Full cohort	-0.19 (-0.33, -0.04)	-17 (-28.2, -4.1)
		No-outlier	-0.18 (-0.33, -0.04)	-16.6 (-27.8, -3.8)
	Model 1	Full cohort	-0.19 (-0.35, -0.03)	-17.2 (-29.2, -3.2)
		No-outlier	-0.18 (-0.34, -0.03)	-16.7 (-28.7, -2.7)
	Model 2	Full cohort	-0.15 (-0.32, 0.02)	-13.8 (-27.2, 2.1)
		No-outlier	-0.14 (-0.3, 0.03)	-13.2 (-26.6, 2.6)
	Model 3	Full cohort	-0.15 (-0.32, 0.02)	-13.5 (-27, 2.4)
		No-outlier	-0.14 (-0.31, 0.03)	-12.9 (-26.4, 3.1)
ALLO-to-Cortisone	Unadjusted	Full cohort	-0.26 (-0.39, -0.13)	-22.6 (-32.1, -11.8)

		No-outlier	-0.25 (-0.38, -0.12)	-22.4 (-31.9, -11.6)
	Model 1	Full cohort	-0.25 (-0.40, -0.11)	-22.4 (-32.7, -10.6)
		No-outlier	-0.25 (-0.39, -0.11)	-22.1 (-32.4, -10.3)
	Model 2	Full cohort	-0.16 (-0.31, -0.01)	-14.7 (-26.6, -0.8)
		No-outlier	-0.16 (-0.30, -0.01)	-14.3 (-26.2, -0.5)
	Model 3	Full cohort	-0.14 (-0.29, 0.01)	-13.2 (-25.2, 0.7)
		No-outlier	-0.14 (-0.29, 0.01)	-12.8 (-24.8, 1)
Cortisol/Cortisone	Unadjusted	Full cohort	-0.08 (-0.14, -0.03)	-8 (-13.3, -2.5)
		No-outlier	-0.09 (-0.14, -0.03)	-8.2 (-13.4, -2.7)
	Model 1	Full cohort	-0.08 (-0.15, -0.02)	-8 (-13.7, -2)
		No-outlier	-0.09 (-0.15, -0.02)	-8.1 (-13.8, -2.1)
	Model 2	Full cohort	-0.04 (-0.10, 0.03)	-3.5 (-9.8, 3.2)
		No-outlier	-0.04 (-0.10, 0.03)	-3.6 (-9.8, 3.1)
	Model 3	Full cohort	-0.02 (-0.09, 0.04)	-2.3 (-8.6, 4.4)
		No-outlier	-0.02 (-0.09, 0.04)	-2.4 (-8.6, 4.3)

Abbreviations: CI – confidence interval.

Model 1: Adjusts for gestational age at blood draw for biomarker measurement, maternal age at enrollment, fetal sex, and prenatal smoking.
Model 2: Model 1 + use of public assistance programs, nativity (U.S.-born yes/no), and marital status.
Model 3: Model 2 + race/ethnicity.

Biomarker concentrations were modeled on the log natural scale. % difference = (exp(estimate) – 1) *100.

Supplemental Table S4. Intra- and Inter-day accuracy and precision from a three-day abbreviated validation of the steroid hormone assay on the analytical platform used in the present study. Quality control samples ($n \geq 5$/day) were analyzed across three independent days at low, medium, and high concentrations. Accuracy is reported as percent nominal concentration recovered, and precision is reported as coefficient of variation (CV%). The abbreviated validation was performed to verify assay performance following transfer of the previously validated method to the current analytical platform.

	Intra-day ranges		Inter-day ranges	
	Accuracy	Precision	Accuracy	Precision
	%	%CV	%	%CV
ALLO	86.4 - 115.5	0.91 - 14.46	97.2 - 108.1	7.66 - 12.72
Pregnanolone	90.5 - 115.3	0.99 - 14.6	97.1 - 110.4	6.81 - 8.87
Progesterone	101.9 - 118.5	1.14 - 7.23	110.3 - 112.7	4.41 - 7.16
Cortisone	88 - 115.6	0.86 - 8.11	86.6 - 111.1	4.01 - 6.86
Cortisol	93.2 - 116.2	1.56 - 7.81	106.6 - 114.9	5.28 - 8.16

Supplemental Table S5. Multiple reaction monitoring (MRM) transitions and optimized mass spectrometer acquisition parameters used for quantification of steroid hormones and internal standards on the AB Sciex API6500 tandem mass spectrometer.

Compound	Q1	Q3	DP (V)	CE (V)	CXP (V)
Allopregnanolone	301.2	283.5	50	19	12
Pregnanolone	301.2	189.2	50	29	12
Progesterone	315.3	109	88	33	7
Cortisone	361	163.3	90	33	12
Cortisol	363.1	121	76	30	8
Allopregnanolone-d5	306.2	288.2	50	19	12
Cortisone-d8	369.2	168.4	102	31	14
Progesterone-d9	324.4	100.1	116	45	14
Cortisone-d4	367.4	121	110	36	22

Q1 and Q3 denote precursor and product ion mass-to-charge ratios (m/z); CE, collision energy; CXP, collision cell exit potential; DP, delustering potential.

Supplemental Figure S1. Heat maps of biomarker correlations using Spearman (Rho) at (**A**) first blood sample (median 17 weeks' gestation) and (**B**) second blood sample (median 27 weeks' gestation) among 237 pregnant individuals in the Healthy Start cohort.

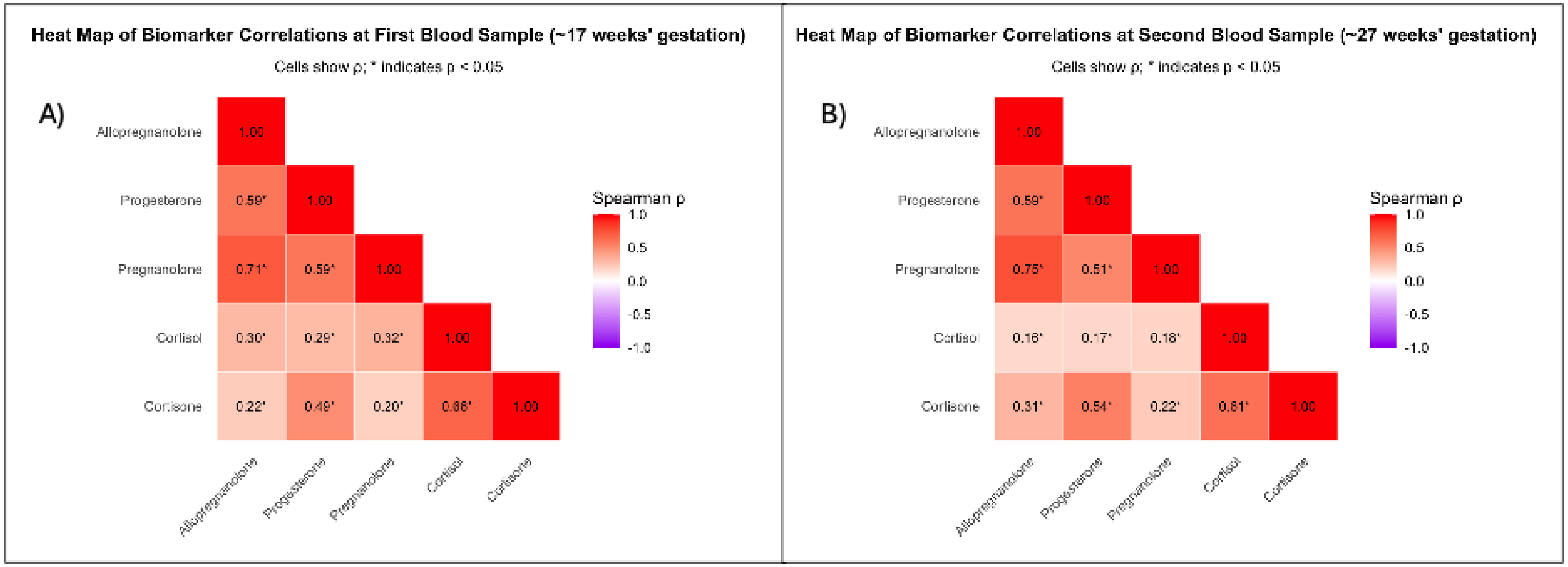

Supplemental Figure S2. Representative extracted ion chromatograms for the quantification of allopregnanolone (ALLO, 3α-hydroxy-5α-pregnan-20-one), progesterone, pregnanolone (3α- hydroxy-5β-pregnan-20-one), cortisol, and cortisone using a liquid chromatography–tandem mass spectrometry (LC–MS/MS) assay validated according to the FDA/ICH M10 guideline.[1] The quantified concentration corresponding to each representative chromatogram is as follows: pregnanolone = 4.3 ng/mL, ALLO = 8.9 ng/mL, progesterone = 39.1 ng/mL, cortisone = 28.1 ng/mL, and cortisol = 160.1 ng/mL.

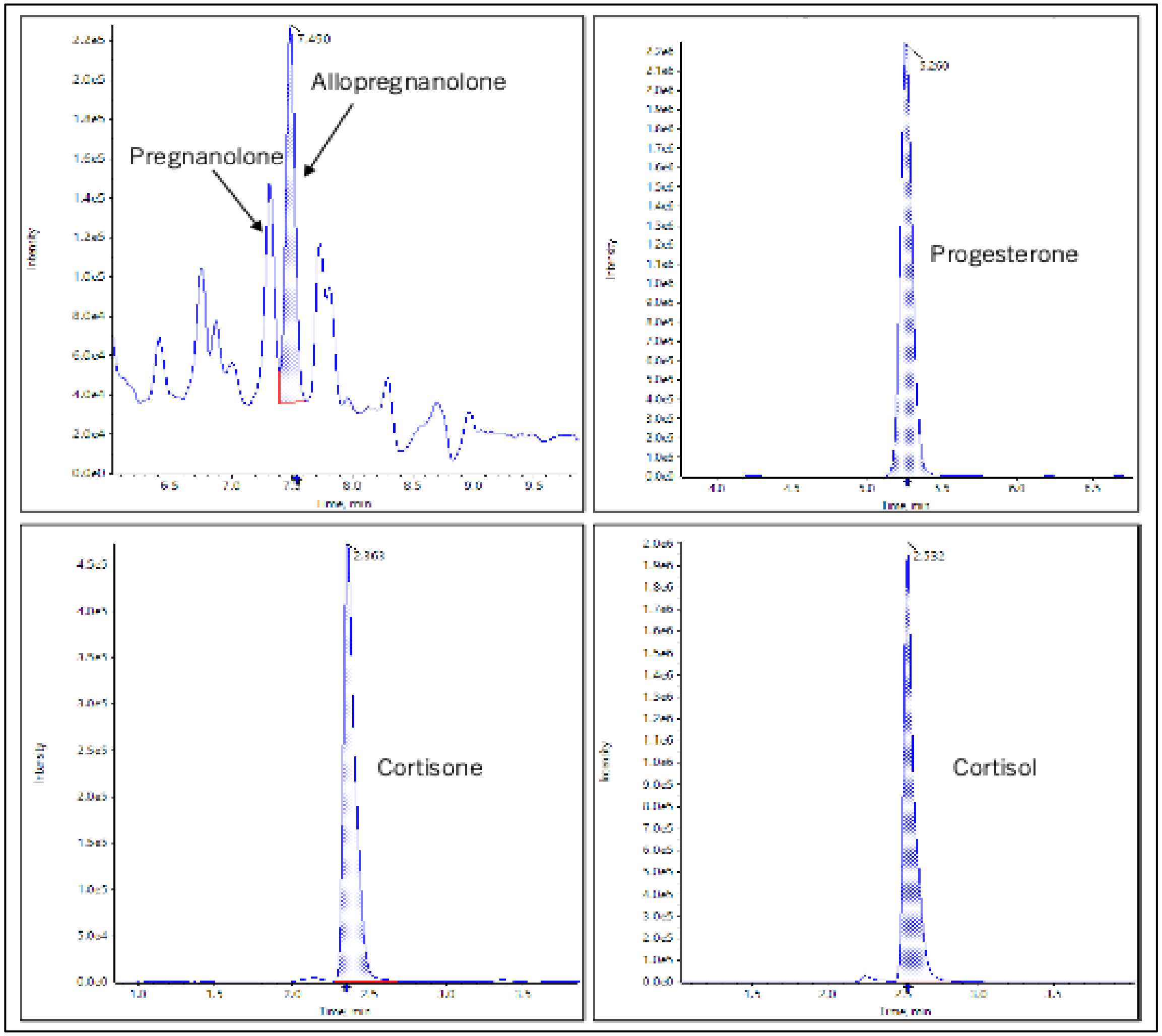

Figure S3. Connections and positions of the switching valve for online extraction (on the left) and backflush (on the right).

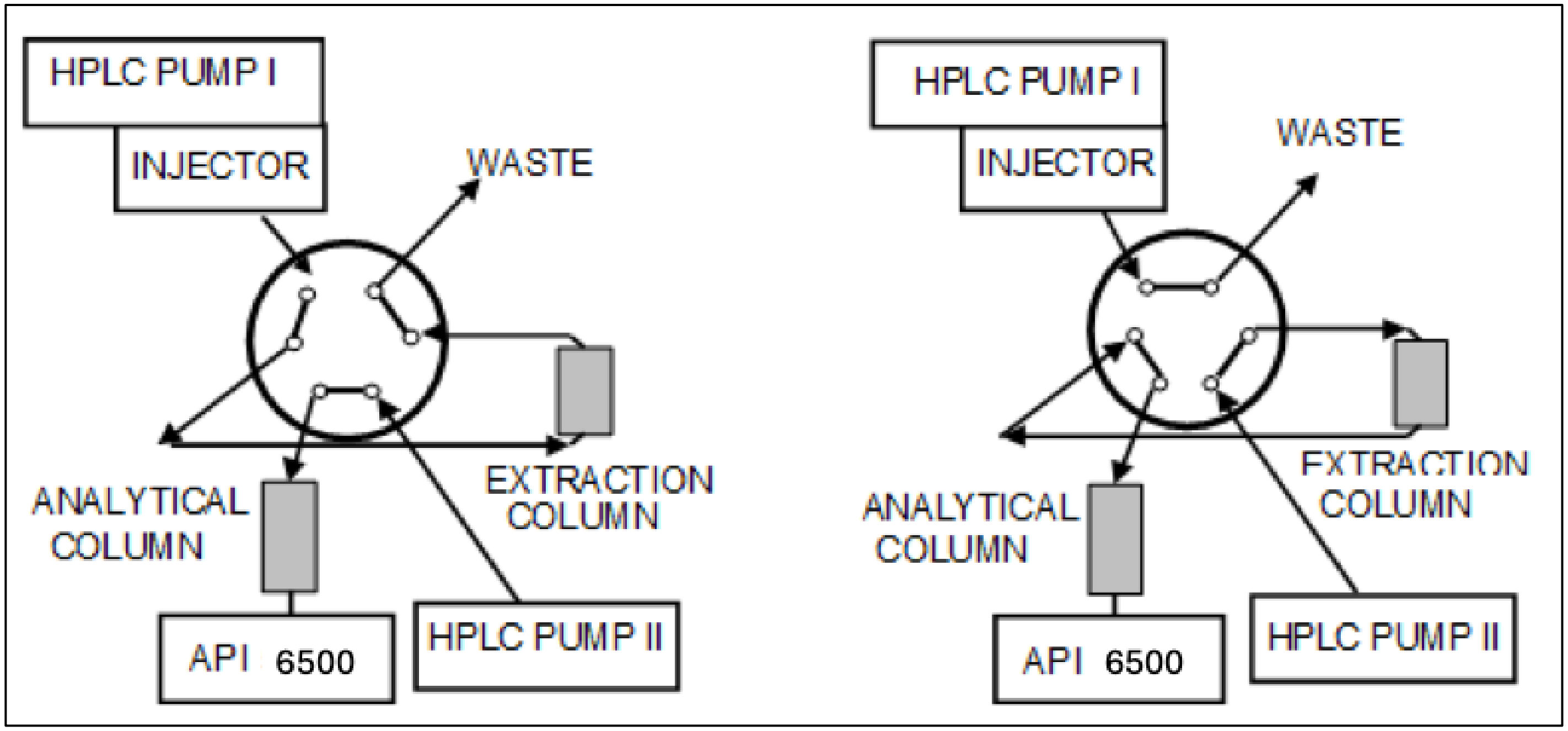

Supplemental Methods:

HPLC-MS/MS parameters were the same as the original published assay.[1] Briefly, frozen serum samples were thawed on ice and 100 μL was aliquoted into microcentrifuge tubes. 400 μL of pure methanol containing 25 ng/mL deuterated internal standards (allopregnanolone-d5, progesterone-d9, cortisol-d4, cortisone-d8, and pregnanolone-d5) was added to precipitate proteins. Allopregnanolone-d5, progesterone-d9, cortisol-d4, and cortisone-d8 were purchased from Sigma-Aldrich (MilliporeSigma), whereas pregnanolone-d5 was purchased from Toronto Research Chemicals (Ontario, Canada). Analytical standards for allopregnanolone and cortisone were obtained from Toronto Research Chemicals, cortisol and progesterone from Cayman Chemical, and pregnanolone from Sigma-Aldrich. Samples were vortexed for 5 minutes and centrifuged at 25,000×g and 4 °C for 10 minutes. The supernatant was transferred into HPLC vials and loaded into the autosampler. Chromatographic separation was performed on an Agilent HPLC system (two binary pumps, thermostated column compartment with an integrated six-port switching valve, and Leap HTC-xt autosampler). The HPLC system was coupled to an AB Sciex API6500 tandem mass spectrometer (Sciex, Concord, ON, Canada) with a turbo ion spray source operated in positive ionization mode. Online extraction was performed using a previously developed column-switching and backflush methodology.[2] This approach concentrates analytes while removing matrix components prior to analytical separation, resulting in improved chromatographic performance and assay throughput.

For online extraction, one hundred microliters (100 μL) of the extracted samples, calibrators and QC samples were injected and loaded onto an Agilent Zorbax XDB-C18, 50 x 2.1 mm (5 µm particle size) HPLC extraction column at 85% LC-MS grade water with 0.1% formic acid and 15% LC-MS grade methanol with 0.1% formic acid at a flow rate increasing from 0.8 mL/minute to 1.5 mL/min within one minute. The retained analytes were then backflushed using a six-port switching valve onto the analytical column (Agilent Poroschell 120, 50 x 4.6 mm, 2.7 µm particle size, EC-C18 material, Agilent Technologies, Santa Clara, CA, USA). The connections and positions of the 6-port switching valve (Rheodyne, Cotati, CA, USA) are shown in **Supplemental Figure S3**.

The API6500 tandem mass spectrometer was used in the positive electrospray ionization mode (+ESI) at an ionization voltage of 5500V in combination with multiple reaction monitoring (for more details, please see **Supplemental Table S5**).

Before analyzing study samples, we conducted a three-day abbreviated validation on this platform pursuant to clinical and industry guidelines.[3] The purpose of this validation was to confirm assay performance following transfer of the previously validated method to a new analytical platform. Acceptance criteria for intra- and inter-day imprecision (coefficient of variation) were ≤20% for all calibrators (≤25% at the lower limit of quantification [LLOQ]) and trueness had to be within ±20% of nominal values (±25% at the LLOQ). Acceptance criteria for calibration curve fit were $r^2 \geq 0.99$ and at least 75% of calibrators had to meet trueness criteria. Quality control (QC) samples in low, medium, and high concentration ranges for each analyte were included. Accuracy and precision of QC samples were within ± 15% of nominal concentrations (± 20% at the LLOQ). Matrix effects and recovery were not re-evaluated during the abbreviated platform validation because the extraction procedure and sample preparation were unchanged from the original validated assay method. On the current platform, the working ranges were 0.39–100 ng/mL for allopregnanolone and pregnanolone, 1.56–400 ng/mL for progesterone and cortisone, and 3.91–1000 ng/mL for cortisol. In our original assay validation, all analytes remained within acceptance criteria after three freeze–thaw cycles and after three months of storage at −80 °C. Long-term frozen-storage stability has been demonstrated for steroid hormones, including progesterone and cortisol, and similar stability is expected for the structurally related neuroactive steroids measured here.[4,5]

References:

1. Mayne G, Bloois ED, Dabelea D, Christians U. Development and validation of an LC-MS/MS assay for the quantification of allopregnanolone and its progesterone-derived isomers, precursors, and cortisol/cortisone in pregnancy. *Anal Bioanal Chem*. 2021;413(21):5427-5438. doi:10.1007/s00216-021-03523-0

2. Klawitter J, Sempio C, Mörlein S, et al. An Atmospheric Pressure Chemical Ionization MS/MS Assay Using Online Extraction for the Analysis of 11 Cannabinoids and Metabolites in Human Plasma and Urine. *Ther Drug Monit*. 2017;39(5):556-564. doi:10.1097/ftd.0000000000000427

3. *M10 Bioanalytical Method Validation and Study Sample Analysis Guidance for Industry*. 2022. Accessed November 1, 2025. https://www.fda.gov/media/162903/download

4. Stroud LR, Solomon C, Shenassa E, et al. Long-term stability of maternal prenatal steroid hormones from the National Collaborative Perinatal Project: Still valid after all these years. *Psychoneuroendocrino*. 2007;32(2):140-150. doi:10.1016/j.psyneuen.2006.11.008

5. Holl K, Lundin E, Kaasila M, et al. Effect of long-term storage on hormone measurements in samples from pregnant women: The experience of the Finnish Maternity Cohort. *Acta Oncol*. 2009;47(3):406-412. doi:10.1080/02841860701592400
